# Increased blood alpha-carotene, all-trans-Beta-carotene and lycopene levels are associated with beneficial changes in heart rate variability: a CVD-stratified analysis in an adult population-based study

**DOI:** 10.1186/s12937-021-00700-w

**Published:** 2021-05-11

**Authors:** Ying Huang, Hong Chen, Yuhao Su, Hualong Liu, Jinzhu Hu, Kui Hong

**Affiliations:** 1 Department of Cardiovascular Medicine, The Second Affiliated Hospital of Nanchang University, No. 1 Minde Road, Donghu District, Nanchang, 330006 Jiangxi China; 2 Jiangxi Key Laboratory of Molecular Medicine, Nanchang, 330006 Jiangxi China

**Keywords:** Carotenoid, Vitamins, Heart rate variability, Cardiovascular diseases, Cross-sectional study

## Abstract

**Background:**

Although the associations of antioxidant micronutrients, such as carotenoids and vitamins, with cardiovascular diseases (CVDs) have been studied extensively, blood concentrations of antioxidant micronutrients and heart rate variability (HRV), which has been proven to be an indicator of cardiac autonomic control, has not been reported. We aimed to explore whether blood concentrations of antioxidant micronutrients, including carotenoids and vitamins, are associated with elevated heart rate variability (HRV (beneficial change) in a cross-sectional analysis.

**Methods:**

Data were obtained from the Midlife in the United States (MIDUS) study that includes a general adult population. A total of 1074 (aged 34–84) individuals were included. Multivariable analyses were performed to investigate the association between main blood carotenoids (total lutein, zeaxanthin, beta-cryptoxanthin, 13-cis-beta-carotene, alpha-carotene, all-trans-beta-carotene and total lycopene) and vitamins A (retinol) and E (gamma-tocopherol and alpha-tocopherol) and HRV after adjustments were made for lifestyle factors and age-related confounders.

**Results:**

Pearson correlation analyses showed that the increased levels of carotenoids and vitamins were positively correlated with higher HRV (all *P* < 0.05). After adjustments were made for age, gender, race, body mass index(BMI), ever-smoker, number of drinking years and exercise, blood alpha-carotene, all-trans-beta-carotene and total lycopene levels were independently associated with higher HRV in the linear regression model (all *P* < 0.05). Sensitivity analysis by adding “ever chronic respiratory diseases” as a covariate suggested that blood concentrations of these three carotenoids were still associated with higher low-frequency (LF)-HRV and high-frequency (HF)-HRV (all *P* < 0.05). Furthermore, stratified analyses suggested that the associations were affected by adding “heart disease” and “hypertension” as covariates.

**Conclusions:**

We provide the first evidence that elevated blood concentrations of alpha-carotene, trans-beta-carotene and lycopene are associated with beneficial changes in HRV in the general population. Daily intake of fruit and vegetables may be beneficial to increase blood carotenoid status and further prevent autonomic dysfunction.

## Introduction

Antioxidant micronutrients, such as carotenoids and vitamins, exist in abundance in fruit and vegetables and have been known to contribute to the body’s defense against adverse inflammation and reactive oxygen species [1, 2]. Numerous epidemiologic studies have demonstrated that a high dietary consumption of fruit and vegetables rich in carotenoids and vitamins with high serum concentrations results in lower risks of cardiovascular diseases (CVDs) [3–6].

CVDs are the leading cause of death and place a high burden of morbidity on the aging populations of industrialized countries [7]. They represent an increasing public health problem because of their high prevalence, need for frequent hospitalization and poor prognosis with detrimental effects at the economic level [8, 9]. Heart rate variability (HRV) is defined as beat-to-beat fluctuations in heart rate that are mainly determined by the activity of the cardiac sympathetic and parasympathetic nervous systems [10]. It has been widely used as a noninvasive and quantitative marker of cardiac autonomic control. As an indicator of autonomic dysfunction, lower HRV has been associated with a significantly increased risk of myocardial infarction (MI), heart failure (HF), hypertension and CVD death in the general population [11–15]. Furthermore, HRV has also been shown to be related to established risk factors for CVDs in many studies [16–18], suggesting that autonomic dysfunction potentially provides a pathway for CVD-related risk factors to be linked to adverse CVD outcomes.

To our knowledge, few previous studies have comprehensively examined the relationship between the blood levels of the main antioxidants and HRV in general population. The aim of the current study was to examine whether higher carotenoid (i.e., total lutein, zeaxanthin, beta-cryptoxanthin, 13-cis-beta-carotene, alpha-carotene, all-trans-beta-carotene and total lycopene) and vitamin A (retinol) and E (gamma-tocopherol and alpha-tocopherol) concentrations were associated with beneficial changes in HRV in a community-based, cross-sectional study of an adult general population.

## Methods

### Study sample

**Fig. 1:**
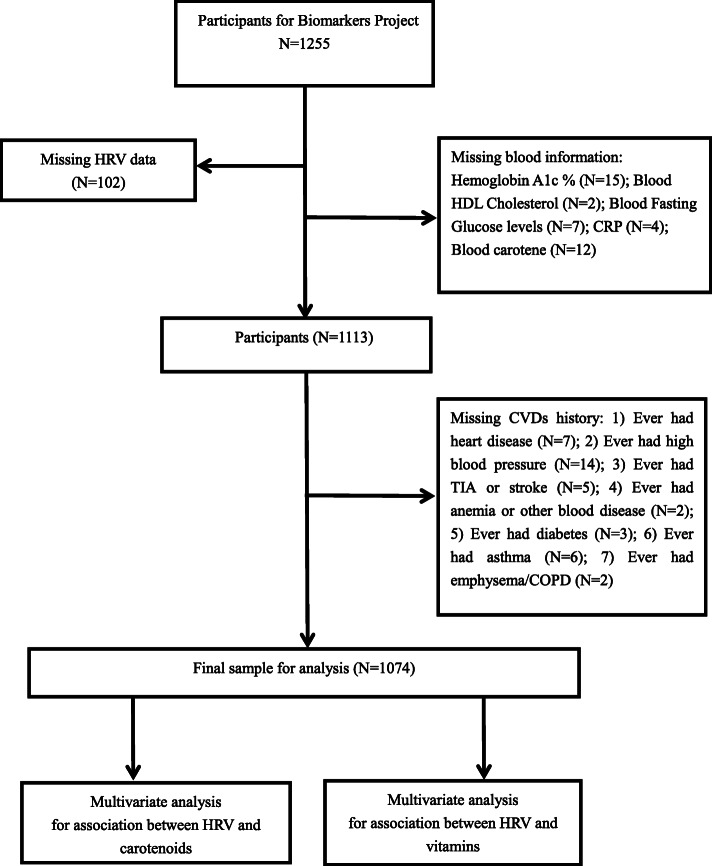
A detailed flow chart of participants included in the study

The data were obtained from 1255 subjects in the MIDUS study, including the behavioral, psychological and social factors in a national sample of adult Americans [19]. Data for the present study are from MIDUS II, a 9-year follow-up of the MIDUS I cohort, conducted between 2004 and 2006. MIDUS II consisted of a self-administered survey of a wide array of social, behavioral and psychological factors, and a biomarker project and data collection were conducted during a 2-day visit to a clinical research center (CRC) at the University of California-Los Angeles, University of Wisconsin or Georgetown University. Blood data were collected from 2004 to 2009 [20]. Each participant was remunerated $200 for participation, and traveling expenses were covered. Clinicians or trained staff evaluated vital signs, morphology, functional capacities and medication usage and performed a physical exam. Medical history was obtained from participants. In summary, a detailed flow chart of the participants included in our study was generated in Fig. 1. After individuals with missing key covariates were excluded, the remaining 1074 individuals were included in the final analysis. In accordance with the Declaration of Helsinki guidelines, the ethics committee of each CRC approved data collection at the three sites, and written consent was obtained from all study participants.

### Measurement of blood

Subjects underwent fasting blood draws prior to breakfast. Samples were sent to the MIDUS Biocore Lab for analysis. The blood concentrations of carotenoids (total lutein, zeaxanthin, beta-cryptoxanthin, 13-cis-beta-carotene, alpha-carotene, all-trans-beta-carotene and total lycopene) and vitamins A and E were assayed using high-performance liquid chromatography at the Mayo Medical Laboratory (Rochester, MN). The coefficient of variation for the blood test was 6.5%. Participants without a blood carotene measurement (*N* = 12) were excluded. Additionally, glycated hemoglobin, fasting glucose, fasting insulin, total cholesterol, triglycerides, low-density lipoprotein (LDL)-cholesterol and high-density lipoprotein (HDL)-cholesterol assays were analyzed at Meriter Labs (Madison, WI) using a Cobas Integra analyzer (Roche Diagnostics, Indianapolis, IN). CRP and creatinine were measured by a BNII nephelometer (Dade Behring Inc., Deerfield, IL).

### Measurement of HRV

Participants were given with a light breakfast without caffeine after an overnight stay at the CRC. After breakfast, the psychophysiology protocol of HRV was carried out. ECG electrodes were placed on the right and left shoulders, as well as in the left lower quadrant. Then, respiration bands were placed around the chest and abdomen, and the finger cuff of the beat-to-beat blood pressure monitor was placed around the middle finger on the nondominant hand. The adjustment of respiration was performed by a spirobag of 800 cc. Data were recorded during an 11-min baseline as part of a more extensive psychophysiology protocol with exposure to challenging stimuli and recovery periods when participants were in the seated position. Here, we collect HRV data from this resting baseline. Analog ECG signals were digitized at 500 Hz by a 16-bit A/D conversion board (National Instruments, Austin, TX) and passed to a microcomputer. The ECG waveform was submitted to an R-wave detection routine implemented by custom-written software, resulting in an RR interval series. Errors in marking R waves were corrected by visual inspection. Ectopic beats were corrected by interpolation. HF-HRV (0.15–0.40 Hz) was computed based on 300-s epochs using an interval method for computing Fourier transforms similar to a previously described method [21]. The mean value of high-frequency (HF)-HRV from the two baseline 300-s epochs was computed. The process was repeated for low-frequency (LF)-HRV (0.04–0.15 Hz). A higher HRV indicated better cardiac autonomic control.

### Covariates

The results in this study needed to be adjusted for because some variables are known for their associations with antioxidant nutrients and HRV. Sociodemographic characteristics and lifestyle factors, including age, sex, race, body mass index (BMI), smoking status, number of drinking years and exercise, were obtained from self-evaluation questionnaires. The questionnaires also collected data on ever having a CVD history, including ever heart disease, ever hypertension, ever transient ischemic attack (TIA) or stroke, ever diabetes mellitus and ever chronic respiratory diseases, including asthma and emphysema/chronic obstructive pulmonary disease (COPD). Race was classified as “white” or “non-white” . Smoking status was also classified as “ever smoker” or “not ever smoker”. Exercise was defined as “whether or not has the frequency of exercises ≥ 3/ week”. Self-reported CVD and chronic respiratory disease histories were dichotomized as “yes” or “no”.

### Statistical analysis

Analyses in the current study were carried out in SPSS 25.0. The normality of the data was analyzed by the Kolmogorov-Smirnov test combined with Q-Q plots. All continuous variables in the study were not normally distributed, so they were expressed as the median (interquartile range [IQR]). The categoric variables were expressed as n (%). Individuals with data missing for a particular variable were removed from our analyses. First, all continuous variables were standardized by using the Z-score and further analyzed by Pearson correlation analysis, which was used to preliminarily evaluate relationships between each of the blood concentrations of 10 antioxidant nutrients and HF and LF-HRV. Multivariate linear regression analysis was further performed to examine the relationships between each antioxidant nutrient and HRV. The significance levels were corrected to account for the 10 associations tested with HF and LF-HRV in each model.

In Model 1, each blood concentration of the 10 antioxidant nutrients was regressed on both HF- and LF-HRV with “age” and “gender” adjusted for. Model 2 was adjusted for age, gender, race and BMI. Model 3 was adjusted by adding ever-smoker, number of drinking years and exercise as covariates. Additionally, to further clarify the effect of chronic respiratory diseases (asthma and emphysema/COPD) on blood concentrations of antioxidant nutrients and their relationship and HRV, we added “chronic respiratory diseases” as a covariate for sensitivity analysis. Finally, stratified analyses were performed by adding “heart disease”, “hypertension”, “TIA or stroke” and “diabetes”. Stratification variables were used to examine whether CVDs impacted the association between blood concentrations of antioxidant nutrients and HRV. A *P* value ≤0.05 was considered statistically significant.

## Results

### The characteristics of all the participants

Table 1 presents sociodemographic characteristics, lifestyle factors and medical history for the 1074 participants included in the study. The median age of all participants in this study was 53.0 years, and 43.3% of them were male. The characteristics of the included participants were as follows: 997 participants (92.8%) were white; 506 participants (47.1%) were ever-smokers; and 829 (77.2%) participants had a frequency of exercise ≥3/week. The median number of drinking years and BMI were 6 and 28.51, respectively. The medians of LF-HRV (0.04–0.15 Hz) and HF-HRV (0.15–0.50 Hz) were 233.20 and 126.60, respectively. The median concentrations of the 10 blood antioxidant nutrients including total lutein, zeaxanthin, beta-cryptoxanthin, 13-cis-beta-carotene, alpha-carotene, all-trans-beta-carotene, total lycopene, gamma-tocopherol, alpha-tocopherol and retinol were 0.229 μmol/L, 0.055 μmol/L, 0.163 μmol/L, 0.054 μmol/L, 0.063 μmol/L, 0.379 μmol/L, 0.409 μmol/L, 3.050 μmol/L, 25.840 μmol/L and 1.660 μmol/L, respectively.

**Table 1:** Characteristics of participants (*N* = 1074)

Variables	N (%) or M (IQR)
Age (years)	53.00 (45.00–62.00)
Gender (male), n (%)	465 (43.3)
Race (white), n (%)	997 (92.8)
BMI (kg/m^2^)	28.51 (25.22–32.85)
Ever smoker, n (%)	506 (47.1)
Number of drinking years	6.00 (2.00–23.00)
Frequency of exercises ≥3/ week, n (%)	829 (77.2)
LF-HRV (0.04–0.15 Hz)	233.20 (104.82–452.88)
HF-HRV (0.15–0.50 Hz)	126.60 (56.23–289.83)
**Ever CVDs history**	
Heart disease, n (%)	99 (9.2)
Hypertension, n (%)	380 (35.4)
TIA or stroke, n (%)	37 (3.4)
Diabetes, n (%)	132 (12.3)
**Ever chronic respiratory diseases**	
Asthma, n (%)	138 (12.8)
emphysema/COPD, n (%)	34 (3.2)
**Blood analysis**	
Blood Total Lutein (umol/L)	0.229 (0.159–0.339)
Blood Zeaxanthin (umol/L)	0.055 (0.039–0.083)
Blood beta-cryptoxanthin (umol/L)	0.163 (0.110–0.254)
Blood 13-cis-beta-carotene (umol/L)	0.054 (0.035–0.090)
Blood alpha-carotene (umol/L)	0.063 (0.031–0.115)
Blood All trans-beta-carotene (umol/L)	0.379 (0.205–0.716)
Blood Total Lycopene (umol/L)	0.409 (0.298–0.553)
Blood gamma-tocopherol (umol/L)	3.050 (1.920–4.960)
Blood alpha-tocopherol (umol/L)	25.840 (20.220–33.530)
Blood Retinol (umol/L)	1.660 (1.310–2.050)
Hemoglobin A1c %	5.82 (5.60–6.20)
Fasting glucose levels mg/dL	96.00 (90.00–105.00)
Fasting insulin levels uIU/mL	10.00 (6.00–17.00)
Total cholesterol (mg/dL)	184.50 (160.00–212.00)
Triglycerides (mg/dL)	106.00 (77.00–156.25)
HDL cholesterol (mg/dL)	53.00 (43.00–66.00)
LDL cholesterol (mg/dL)	102.00 (81.00–128.00)
Creatinine (mg/dL)	0.80 (0.70–1.00)
C-reactive protein (ug/mL)	1.39 (0.68–3.60)

M (IQR) for non-normally distributed variables, and n (%) for categoric variables

*BMI* body mass index; *LF-HRV* Low frequency-heart rate variability; *HF-HRV* High frequency heart rate variability; *TIA* transient ischemic attack; *COPD* chronic obstructive pulmonary disease; *HOMA-IR* homeostasis model assessment of insulin resistance; *HDL* high density lipoprotein; *LDL* low density lipoprotein

### Elevated blood carotenoid levels were positively associated with higher HRV by using Pearson correlation analysis

Our results with Pearson correlation analyses showed that elevated blood concentrations of carotenoids, including total lutein, zeaxanthin, beta-cryptoxanthin, 13-cis-beta-carotene, alpha-carotene, all-trans-beta-carotene, total lycopene and vitamins, including gamma-tocopherol, alpha-tocopherol and retinol, were associated with higher LF-HRV and HF-HRV (all *P* < 0.05, Table 2). The single factor correlation analysis preliminarily implied that the blood concentrations of the 10 antioxidant nutrients were closely related to beneficial changes in HRV.

**Table 2:** Bivariate correlations Using Standardized Variables (*N* = 1074)

Variables	LF-HRV		HF-HRV	
	Sβ	*P* Value	Sβ	*P* Value
Blood Total Lutein (umol/L)	0.068	0.016	0.068	0.016
Blood Zeaxanthin (umol/L)	0.068	0.016	0.068	0.016
Blood beta-cryptoxanthin (umol/L)	0.067	0.018	0.067	0.018
Blood 13-cis-beta-carotene (umol/L)	0.066	0.020	0.065	0.021
Blood alpha-carotene (umol/L)	0.077	0.007	0.076	0.007
Blood All trans-beta-carotene (umol/L)	0.111	< 0.001	0.110	< 0.001
Blood Total Lycopene (umol/L)	0.086	0.002	0.086	0.002
Blood gamma-tocopherol (umol/L)	0.061	0.032	0.061	0.029
Blood alpha-tocopherol (umol/L)	0.070	0.013	0.070	0.013
Blood Retinol (umol/L)	0.068	0.016	0.068	0.016

*LF-HRV* Low frequency-heart rate variability; *HF-HRV* High frequency heart rate variability

Pearson correlation analysis was used

### Blood alpha-carotene, all-trans-beta-carotene and total lycopene levels were positively and independently associated with beneficial changes in HRV by using multivariate linear regression analysis

To further analyze the independent associations of these 10 antioxidant nutrients and HRV, multivariate linear regression analysis was performed (Table 3). Our results indicated that elevated blood concentrations of carotenoids, including total lutein, zeaxanthin, beta-cryptoxanthin, 13-cis-beta-carotene, alpha-carotene, all-trans-beta-carotene, total lycopene and vitamins, including gamma-tocopherol, alpha-tocopherol and retinol, were associated with higher LF-HRV and HF-HRV after adjusting for age and gender (all *P* < 0.05, Model 1). In Model 2, however, only blood alpha-carotene, all-trans-beta-carotene and total lycopene levels were associated with higher LF-HRV and HF-HRV after adjustments were made for age, gender, race and BMI. Model 3 suggested that these three carotenoids were still significantly related to a higher LF-HRV and HF-HRV after continuing to add ever-smoker, number of drinking years and exercise into Model 2 (all *P* < 0.05). These results suggested that blood alpha-carotene, all-trans-beta-carotene and total lycopene levels are independently associated with beneficial changes in HRV after adjusting for sociodemographic characteristics and lifestyle factors.

**Table 3:** Multiple linear regression analysis for relationship between blood carotenoid levels and HRV

	LF-HRV			HF-HRV		
Variables	Sβ	95% CI	*P* Value	Sβ	95% CI	*P* Value
**Model 1**						
Blood Total Lutein	0.067	0.013–0.122	0.016	0.067	0.012–0.122	0.016
Blood Zeaxanthin	0.067	0.012–0.122	0.016	0.067	0.012–0.122	0.016
Blood beta-cryptoxanthin	0.066	0.011–0.121	0.018	0.066	0.011–0.121	0.018
Blood 13-cis-beta-carotene	0.067	0.013–0.122	0.016	0.067	0.012–0.121	0.017
Blood alpha-carotene	0.077	0.022–0.132	0.006	0.076	0.022–0.131	0.006
Blood All trans-beta-carotene	0.114	0.060–0.169	< 0.001	0.113	0.059–0.168	< 0.001
Blood Total Lycopene	0.085	0.031–0.140	0.002	0.085	0.031–0.140	0.002
lood gamma-tocopherol	0.064	0.010–0.119	0.021	0.065	0.010–0.120	0.020
Blood alpha-tocopherol	0.066	0.011–0.121	0.018	0.066	0.011–0.121	0.018
Blood Retinol	0.066	0.011–0.121	0.018	0.066	0.011–0.120	0.018
**Model 2**						
Blood Total Lutein	0.049	−0.007-0.104	0.089	0.048	− 0.008-0.104	0.091
Blood Zeaxanthin	0.048	−0.008-0.104	0.090	0.048	−0.008-0.104	0.092
Blood beta-cryptoxanthin	0.047	−0.008-0.103	0.096	0.047	−0.009-0.103	0.098
Blood 13-cis-beta-carotene	0.051	−0.004-0.107	0.071	0.050	−0.005-0.105	0.075
Blood alpha-carotene	0.060	0.005–0.116	0.034	0.060	0.004–0.115	0.036
Blood All trans-beta-carotene	0.101	0.046–0.156	< 0.001	0.100	0.045–0.155	< 0.001
Blood Total Lycopene	0.068	0.012–0.124	0.017	0.068	0.012–0.123	0.017
Blood gamma-tocopherol	0.045	−0.011-0.101	0.112	0.046	−0.010-0.102	0.106
Blood alpha-tocopherol	0.047	−0.009-0.103	0.098	0.047	−0.009-0.103	0.099
Blood Retinol	0.047	−0.009-0.103	0.098	0.047	−0.009-0.103	0.100
**Model 3**						
Blood Total Lutein	0.045	−0.011-0.101	0.116	0.045	−0.011-0.101	0.116
Blood Zeaxanthin	0.045	−0.011--0.101	0.118	0.044	−0.012-0.100	0.121
Blood beta-cryptoxanthin	0.044	−0.012-0.100	0.125	0.043	−0.013-0.100	0.128
Blood 13-cis-beta-carotene	0.047	−0.008-0.103	0.095	0.047	−0.009-0.102	0.100
Blood alpha-carotene	0.057	0.002–0.113	0.044	0.057	0.001–0.112	0.047
Blood All trans-beta-carotene	0.098	0.043–0.153	0.001	0.097	0.042–0.152	0.001
Blood Total Lycopene	0.065	0.009–0.121	0.023	0.064	0.009–0.120	0.024
Blood gamma-tocopherol	0.041	−0.015-0.098	0.149	0.042	−0.014-0.098	0.143
Blood alpha-tocopherol	0.044	−0.012-0.100	0.124	0.044	−0.012-0.100	0.127
Blood Retinol	0.044	−0.012-0.100	0.126	0.043	−0.013-0.099	0.130

Model 1: Adjusted for age and gender

Model 2: Adjusted for age, gender, race and BMI

Model 3: Adjusted for age, gender, race, BMI, ever smoker, number of drinking years and exercise

*LF-HRV* Low frequency-heart rate variability; *HF-HRV* High frequency heart rate variability

Furthermore, the associations between these three carotenoid levels and HRV were determined using sensitivity analysis by adding “respiratory diseases” as a covariate (Table 4), which has been shown to be associated with changed blood levels of antioxidant nutrients [22, 23]. The results showed that blood alpha-carotene, all-trans-beta-carotene and total lycopene levels were still independently associated with higher LF-HRV and HF-HRV (all *P* < 0.05). These findings suggested that elevated blood alpha-carotene, all-trans-beta-carotene and total lycopene levels were significantly increased with beneficial changes in HRV.

**Table 4:** Sensitivity analysis for relationship between blood carotenoid levels and HRV

	LF-HRV			HF-HRV		
Variables	Sβ	95% CI	*P* Value	Sβ	95% CI	*P* Value
**Model 1**						
Blood alpha-carotene	0.078	0.023–0.132	0.005	0.077	0.023–0.132	0.006
Blood All trans-beta-carotene	0.115	0.060–0.169	< 0.001	0.114	0.060–0.168	< 0.001
Blood Total Lycopene	0.086	0.032–0.141	0.002	0.086	0.031–0.140	0.002
**Model 2**						
Blood alpha-carotene	0.061	0.005–0.117	0.031	0.060	0.005–0.116	0.033
Blood All trans-beta-carotene	0.102	0.047–0.157	< 0.001	0.101	0.046–0.156	< 0.001
Blood Total Lycopene	0.069	0.013–0.124	0.016	0.068	0.012–0.124	0.016
**Model 3**						
Blood alpha-carotene	0.058	0.002–0.113	0.042	0.057	0.001–0.113	0.044
Blood All trans-beta-carotene	0.098	0.043–0.154	< 0.001	0.098	0.042–0.153	0.001
Blood Total Lycopene	0.065	0.010–0.121	0.022	0.065	0.009–0.121	0.022

Model 1: Adjusted for age, gender and ever chronic respiratory diseases

Model 2: Adjusted for age, gender, race, BMI and ever chronic respiratory diseases

Model 3: Adjusted for age, gender, race, BMI, ever smoker, number of drinking years, exercise and ever chronic respiratory diseases

*LF-HRV* Low frequency-heart rate variability; *HF-HRV* High frequency heart rate variability

### Stratified analyses of the associations between blood alpha-carotene, all-trans-beta-carotene and total lycopene levels and HRV

Table 5 presents stratified analyses performed by using CVD history including “ever heart disease”, “ever hypertension”, “ever TIA or stroke” and “ever diabetes” separately as a stratification variable to assess the associations between blood concentrations of alpha-carotene, all-trans-beta-carotene and total lycopene and HRV. Interestingly, in the participants with heart disease and hypertension, we found that the independent associations between blood carotenoid levels (alpha-carotene, all-trans-beta-carotene and total lycopene) and HRV disappeared (all *P* > 0.05), while the associations of the three blood carotenoid levels with HRV were much stronger in the participants without heart disease and hypertension (all *P* < 0.05). Additionally, in the participants with TIA or stroke and diabetes, however, our results suggested that although the correlation between carotenoid levels and HRV was much weaker in the population with TIA or stroke and diabetes, independent associations between the three carotenoid levels and HRV still existed (all *P* < 0.05; Table 5). These results suggested that the associations between blood concentrations of alpha-carotene, all-trans-beta-carotene and total lycopene and HRV were affected by heart disease and hypertension, while stroke and diabetes had little effect on associations.

**Table 5:** Stratified analysis for association between blood carotenoid levels and HRV by adding “ever CVDs” as covariates

	LF-HRV			HF-HRV		
Variables	Sβ	95% CI	*P* Value	Sβ	95% CI	*P* Value
**Heart disease (yes)**						
Blood alpha-carotene	0.045	−0.019-0.082	0.128	0.044	−0.020-0.081	0.128
Blood All trans-beta-carotene	0.049	−0.015-0.122	0.099	0.048	−0.016-0.121	0.100
Blood Total Lycopene	0.043	−0.021-0.100	0.130	0.042	−0.022-0.099	0.130
**Heart disease (no)**						
Blood alpha-carotene	0.069	0.004–0.141	0.021	0.068	0.003–0.140	0.023
Blood All trans-beta-carotene	0.117	0.046–0.173	< 0.001	0.116	0.045–0.172	< 0.001
Blood Total Lycopene	0.076	0.012–0.128	0.019	0.075	0.011–0.127	0.019
**Hypertension (yes)**						
Blood alpha-carotene	0.047	−0.018-0.084	0.122	0.046	−0.019-0.083	0.122
Blood All trans-beta-carotene	0.051	−0.013-0.125	0.092	0.050	−0.014-0.124	0.100
Blood Total Lycopene	0.044	−0.020-0.101	0.129	0.043	−0.021-0.100	0.129
**Hypertension (no)**						
Blood alpha-carotene	0.067	0.002–0.138	0.025	0.066	0.001–0.137	0.026
Blood All trans-beta-carotene	0.114	0.044–0.170	< 0.001	0.113	0.043–0.169	< 0.001
Blood Total Lycopene	0.075	0.011–0.126	0.020	0.075	0.011–0.126	0.020
**TIA or stroke (yes)**						
Blood alpha-carotene	0.057	0.001–0.112	0.044	0.056	0.001–0.112	0.045
Blood All trans-beta-carotene	0.097	0.041–0.153	< 0.001	0.096	0.040–0.152	0.002
Blood Total Lycopene	0.064	0.0009–0.120	0.023	0.064	0.008–0.120	0.023
**TIA or stroke (no)**						
Blood alpha-carotene	0.059	0.002–0.114	0.041	0.058	0.002–0.113	0.042
Blood All trans-beta-carotene	0.099	0.044–0.156	< 0.001	0.099	0.043–0.155	< 0.001
Blood Total Lycopene	0.066	0.003–0.122	0.020	0.066	0.002–0.122	0.020
**Diabetes (yes)**						
Blood alpha-carotene	0.056	0.001–0.111	0.046	0.055	0.001–0.110	0.047
Blood All trans-beta-carotene	0.095	0.040–0.151	< 0.001	0.094	0.039–0.151	0.002
Blood Total Lycopene	0.063	0.008–0.119	0.027	0.063	0.008–0.118	0.027
**Diabetes (no)**						
Blood alpha-carotene	0.060	0.004–0.115	0.040	0.059	0.003–0.114	0.042
Blood All trans-beta-carotene	0.101	0.045–0.157	< 0.001	0.100	0.044–0.156	< 0.001
Blood Total Lycopene	0.067	0.011–0.126	0.019	0.067	0.010–0.126	0.019

Adjusted for age, gender, race, BMI, ever smoker, number of drinking years, exercise and ever chronic respiratory diseases

*LF-HRV* Low frequency-heart rate variability; *HF-HRV* High frequency heart rate variability

## Discussion

In this community-based, cross-sectional analysis of an adult population, we found that elevated blood alpha-carotene, all-trans-beta-carotene and total lycopene levels were strongly associated with beneficial changes in HRV, which may lead to a reduced risk of CVDs through favorable changes in cardiac autonomic function.

The development of autonomic dysfunction in adult population is accompanied by a four-fold higher risk of cardiac autonomic neuropathy, which has been associated with increased CVD mortality [12–14]. HRV has been shown to have direct independent consequences in terms of morbidity and mortality in patients with CVDs, including coronary artery disease, heart failure, stroke and hypertension [11–15]. Although many studies have shown that adequate intake of carotene is beneficial to the prevention of cardiovascular incidence or mortality [24–26], few studies have reported the associations between blood levels of antioxidant micronutrients and HRV that can assess cardiac autonomic function. Previous studies have reported a reduced risk of coronary heart disease, stroke, CVDs and CVD mortality with a high intake of carotenoids [25–33]. Blood concentrations of lycopene were correlated with tomato and tomato juice intake [31, 33–35] and the intake of tomatoes has also been inversely associated with coronary heart disease, although associations with other outcomes were less clear [34]. Our results suggested that strong associations between carotenoids and HRV did exist in our results, which was consistent with previous evidence [3–6, 11–15]. We also found that elevated serum total lycopene levels were associated with a beneficial change in HRV. This might be explained by the mechanism by which antioxidant micronutrients contribute to the body’s defense against adverse inflammation and reactive oxygen species [1, 2], which have been strongly associated with a reduced risk of CVDs through favorable changes in cardiac autonomic function [16–21].

Interestingly, in the participants with heart disease and hypertension, we found that an independent association between blood carotenoid levels (alpha-carotene, all-trans-beta-carotene and total lycopene) and HRV disappeared (all *P* > 0.05), while these associations were much stronger in the participants without heart disease and hypertension (all *P* < 0.05). Although there is existing evidence about associations of reduced HRV with an increased risk of CVDs and death [11–15], the results of the stratification differences may be partly explained by the intrinsic impairment in autonomic function caused by coronary heart disease (CAD) or other heart diseases resulting in disruptions in the significant relationships. However, the associations between blood alpha-carotene, all-trans-beta-carotene and total lycopene levels and HRV were not affected by diabetes and TIA or stroke. Some studies have suggested that patients with diabetes and TIA or stroke tend to have lower HRV [11–15], a significant association still existed in our study. One possible explanation is that stroke has little effect on autonomic function. Advanced diabetes can cause neuropathy, but early diabetes has little effect on autonomic nerve function. In summary, the differences observed with the stratification of these variables need to be further confirmed in future studies.

Some previous studies have suggested that chronic vitamin A and E administration improved HRV and reduced the risk of CVDs in general populations [36–38]. Potential mechanisms that improve HRV by increasing vitamin intake may reduce the risk of CVDs by antiarrhythmic, hypotriglyceridemic and antithrombogenic effects, inhibition of atherosclerotic plaque growth and promotion of nitric oxide–induced endothelial relaxation [39]. However, we did not find a significant associations between HRV and blood retinol, alpha-tocopherol or gamma-tocopherol after adjusting for enough confounding factors, which is inconsistent with previous studies showing that high concentrations of vitamins were associated with a reduced risk of CVDs. The inconsistent results may be at least partly explained by the different study designs used, the different hypotheses being investigated, the different confounding factors included and the inherent difficulties of obtaining epidemiologic measurements of intricate factors. For example, HRV measurements can vary according to psychological, physiological, or environmental factors, especially in individuals across the age range. Additionally, different questionnaires assessing sociodemographic characteristics and lifestyle factors may also contribute to some of these variations.

Important strengths in this study include the community-based design with a large number of participants, providing sufficient power to detect small changes in HRV measures. Although not all subjects had repeated measures of HRV, more than one-half of subjects had repeated measures that allowed us to adjust for subject-specific variations in HRV. We treated age, gender, race, BMI, smoking status, drinking status, exercise and other variables as confounding factors, affording better control for them relative to the cross-sectional analysis. Previous studies have only analyzed the relationship between the intake of carotenoids and/or carotenoid-related food and HRV [40]. We collected relevant biochemistry data, including levels of 7 main serum carotenoids, in this study, which is a more intuitive and reliable way to determine the relationship between the blood concentration of carotenoids and HRV. Additionally, given the potential link between carotenoids and CVDs, the associations between blood carotenoids and HRV were further determined using stratified analysis by adding “CVDs” as a covariate. The results still showed that blood alpha-carotene, all-trans-beta-carotene and total lycopene levels were independently associated with higher HRV.

This study has several limitations. First, this was a cross-sectional study and it was not possible to make causal inferences between carotenoids and HRV. Second, to participate in this study, all participants needed to be healthy enough to go to a MIDUS study research center. Thus it may lead to potential selection bias. Third, we could not exclude the possibility that the observed associations were attributable to other factors that correlate with blood carotenoids and HRV. Because HRV is a sensitive marker, it can be influenced by some environmental factors. The measurement of serum carotenoid levels is also affected by patients’ physiological state, measurement and methods. Fourth, the stratified variables mainly relied on the questionnaire survey provided by the participants, which is not a good method to determine the history of CVDs. This may have caused some deviation in the stratified results. Finally, we could not rule out uncontrolled confounding by other unmeasured variables, and some of our findings may have occurred by chance.

## Conclusions

Our study provides the first evidence that elevated serum alpha-carotene, all-trans-beta-carotene and total lycopene levels are associated with beneficial effects on cardiac autonomic dysfunction in the general population, which may explain why a healthy diet including sufficient carotenoids is an important way to prevent or improve CVDs, even if more research is needed.

## Data Availability

All data generated or analyzed during this study were obtained from MIDUS study.
